# Supplementary material for: EXercise to Prevent frailty and Loss Of independence in insulin treated older people with DiabetEs (EXPLODE): protocol for a feasibility randomised controlled trial (RCT)
Source: BMJ Open. 2021 Dec 7;11(12):e048932. doi: 10.1136/bmjopen-2021-048932 (PMC8655574; doi:10.1136/bmjopen-2021-048932)
Supplement: Author's manuscript [file bmjopen-2021-048932.draft_revisions.pdf]

BMJ Open is committed to open peer review. As part of this commitment we make the peer review history of every article we publish publicly available.

When an article is published we post the peer reviewers' comments and the authors' responses online. We also post the versions of the paper that were used during peer review. These are the versions that the peer review comments apply to.

The versions of the paper that follow are the versions that were submitted during the peer review process. They are not the versions of record or the final published versions. They should not be cited or distributed as the published version of this manuscript.

BMJ Open is an open access journal and the full, final, typeset and author-corrected version of record of the manuscript is available on our site with no access controls, subscription charges or pay-per-view fees (http://bmjopen.bmj.com).

If you have any questions on BMJ Open's open peer review process please email
info.bmjopen@bmj.com

BMJ Open

EXercise to Prevent frailty and Loss Of independence in insulin treated older people with DiabetEs (EXPLODE): protocol for a feasibility randomised controlled trial (RCT)

Journal:	*BMJ Open*
Manuscript ID	bmjopen-2021-048932
Article Type:	Protocol
Date Submitted by the Author:	13-Jan-2021
Complete List of Authors:	Stocker, Rachel; Newcastle University<br>Shaw, James; Newcastle University<br>Taylor, Guy S; Newcastle University<br>Witham, Miles; Newcastle University, NIHR Newcastle Biomedical Research Centre<br>West, Daniel J; Newcastle University, Population Health Sciences Institute
Keywords:	DIABETES & ENDOCRINOLOGY, SPORTS MEDICINE, Clinical trials < THERAPEUTICS, GERIATRIC MEDICINE

SCHOLARONE™
Manuscripts

I, the Submitting Author has the right to grant and does grant on behalf of all authors of the Work (as defined in the below author licence), an exclusive licence and/or a non-exclusive licence for contributions from authors who are: i) UK Crown employees; ii) where BMJ has agreed a CC-BY licence shall apply, and/or iii) in accordance with the terms applicable for US Federal Government officers or employees acting as part of their official duties; on a worldwide, perpetual, irrevocable, royalty-free basis to BMJ Publishing Group Ltd ("BMJ") its licensees and where the relevant Journal is co-owned by BMJ to the co-owners of the Journal, to publish the Work in this journal and any other BMJ products and to exploit all rights, as set out in our licence.

The Submitting Author accepts and understands that any supply made under these terms is made by BMJ to the Submitting Author unless you are acting as an employee on behalf of your employer or a postgraduate student of an affiliated institution which is paying any applicable article publishing charge ("APC") for Open Access articles. Where the Submitting Author wishes to make the Work available on an Open Access basis (and intends to pay the relevant APC), the terms of reuse of such Open Access shall be governed by a Creative Commons licence – details of these licences and which Creative Commons licence will apply to this Work are set out in our licence referred to above.

Other than as permitted in any relevant BMJ Author's Self Archiving Policies, I confirm this Work has not been accepted for publication elsewhere, is not being considered for publication elsewhere and does not duplicate material already published. I confirm all authors consent to publication of this Work and authorise the granting of this licence.

For peer review only

EXercise to Prevent frailty and Loss Of independence in insulin treated older people with DiabetEs (EXPLODE): protocol for a feasibility randomised controlled trial (RCT)

For peer review only

Rachel Stocker[1]†, James A Shaw[2], Guy S Taylor[1], Miles D Witham[2], Daniel J West[1]†

† indicates joint corresponding author

[1] Population Health Sciences Institute, Faculty of Medical Sciences, Newcastle University

[2] Translational and Clinical Research Institute, Faculty of Medical Sciences, Newcastle University

Corresponding authors: Dr Rachel Stocker, rachel.stocker@newcastle.ac.uk and Dr Daniel West, daniel.west@newcastle.ac.uk

Abstract

For peer review only - http://bmjopen.bmj.com/site/about/guidelines.xhtml

Introduction

There are 3.9m people in the UK with diabetes. Sarcopenia, increased frailty, and loss of independence are often unappreciated complications of diabetes. Resistance exercise shows promise in reducing these complications in non-elderly diabetes patients. The aim of this feasibility randomised controlled trial is to (1) characterise the physical function, cardiovascular health, and the health and wellbeing of older adults with insulin treated diabetes and mild frailty, (2) to test the feasibility and efficacy of a four-week resistance exercise training programme in improving these parameters, (3) to test the feasibility of recruiting and randomising the target participant group to a trial of resistance training.

Methods and analysis

Thirty adults aged ≥60 years with insulin treated diabetes mellitus (type 1 or 2), and thirty without, all with mild frailty (3-4 on the Rockwood Frailty Scale) will be recruited. Each group will be age, gender, and frailty matched. All will complete blood, cardiovascular, and physical function testing. Each group will be randomised 1:1 to a 4-week semi-supervised resistance training programme, designed to increase muscle mass and strength, or to usual care, defined as their regular

For peer review only - http://bmjopen.bmj.com/site/about/guidelines.xhtml

physical activity, for 4 weeks. All participants will then repeat testing. Primary outcomes include recruitment rate, attrition rate, intervention fidelity and acceptability, and adherence to the training programme. A subset of participants in all trial arms will be interviewed before and after the training programme to understand experiences of resistance training, impact on health, and living with diabetes (where relevant) as they have aged. Analyses will include descriptive statistics and qualitative thematic analysis.

Ethics and dissemination

The North East-Newcastle & North Tyneside 2 Research Ethics Committee (20/NE/0178) approved the study. Outputs will include feasibility data to support funding applications for a future definitive trial, conference and patient and public involvement presentations, and peer-reviewed publications.

Trial registration

Current Controlled Trials: ISRCTN13193281.

For peer review only - http://bmjopen.bmj.com/site/about/guidelines.xhtml

Strengths and limitations of this study

- This is a novel study to examine the feasibility of carrying out a larger trial of a gym-based resistance exercise training programme with older, mildly frail adults with insulin-treated diabetes.
- This is a single-site feasibility randomised controlled trial which will be carried out in the North East of England.
- A mixed-methods approach will explore the impact of the training programme on various clinical outcomes.
- Pre- and post- intervention qualitative interviews will generate a better understanding of the barriers and facilitators to resistance-based exercise for this important patient group, where early intervention is key.

For peer review only

For peer review only - http://bmjopen.bmj.com/site/about/guidelines.xhtml

Introduction

Background

There are around 425 million people with diabetes worldwide, and by 2040 it is predicted that 1 in 10 people globally with have diabetes.[1] In the UK there are around 4 million people living with diabetes[2] (90% Type 2, 8% Type 1, 2% other) with a further 1 million with undiagnosed diabetes. The prevalence of diabetes increases sharply with age, with 17.4% of those aged over 65 having diabetes, compared to 2% of those aged 16 to 44 in England.[3] All of those with type 1 diabetes require insulin treatment, and most of those with type 2 diabetes will eventually also require insulin treatment.[4 5] Diabetes represents >10% of the NHS budget in direct treatment costs.[6] Modern advances in diabetes treatment mean that people with diabetes are living longer[7], even with the presence of the micro- and macrovascular disease associated with long-term diabetes.

With an ageing population of people living with diabetes, it is important that strategies for improving both health, quality of life, and reducing treatment burden

For peer review only - http://bmjopen.bmj.com/site/about/guidelines.xhtml

are identified. Long-term insulin treatment for diabetes is, however, associated with detrimental effects to health including hypoglycaemia and weight gain[8], and may adversely affect muscle health.[9] Sarcopenia in particular leads to an increased risk of frailty, falls, physical disability, chronic metabolic disease, and mortality.[10 11] The prevalence of frailty in older people with diabetes has been reported as ~32–48%, which is significantly higher than that of 5–10% in older persons without diabetes.[12 13] Recent studies have also demonstrated an increased risk of osteoporosis and fracture in older people with diabetes, compared to age matched non-diabetes controls.[14 15] Thus long-term diabetes and long-term insulin treatment, hypoglycaemia and age related physical decline, may carry an additional burden for those living with insulin treated diabetes in later life.

Physical activity and exercise interventions have been shown to improve outcomes associated with frailty and sarcopenia (such as muscle mass, muscle force production, cardiorespiratory fitness).[16] However, these interventions are not straightforward in people with insulin treated diabetes due to the risk of hypoglycaemia.[17] Aerobic exercise increases insulin sensitivity, changes the absorption and action of the injected insulin, and increases metabolic rate

For peer review only - http://bmjopen.bmj.com/site/about/guidelines.xhtml

dramatically.[18 19] Under these conditions, in a person without diabetes, the insulin concentrations would drop dramatically during and after exercise, however, in those with diabetes this is not possible (as the insulin is injected), and thus increases the risk of dangerously low blood glucose occurring. Nearly two-thirds of those with type 1 diabetes do not engage in any physical activity[20], commonly due to fear of exercise-induced hypoglycaemia.[21]

Resistance based exercise, i.e. repeated intense muscle contractions of isolated parts of the body against a fixed load, is associated with less fluctuation in blood glucose than aerobic exercise.[22-24] Resistance exercise is a potent stimulus for improving 1) muscle mass, 2) muscle strength and power, 3) bone health, and 4) physical function, such as stair climbing.[25 26] Existing studies have shown resistance training to be a useful exercise modality in non-elderly, non-frail type 1 and type 2 diabetes patients.[27-29] It is also the modality of exercise with the most evidence for improving outcomes in older adults with sarcopenia or frailty,[30 31] and generally well tolerated by this group.[32] However, data for its use in older adults with diabetes is scant. As resistance training potentially carries less risk of blood glucose fluctuation to those with diabetes, due to differing hormonal responses to aerobic exercise, it

For peer review only - http://bmjopen.bmj.com/site/about/guidelines.xhtml

has potential to be a preferred modality of exercise for this group, and may help sustain long-term engagement.[22-24]

Given the increasing ageing diabetes population, and the increased risk of sarcopenia and frailty in this group, it is important to provide lifestyle related interventions, such as resistance training, to improve the quality of life of older people living with diabetes.[33] At present there is limited information on the physical function of older people with diabetes comparted to those without diabetes, as well as how acceptable or feasible a resistance training intervention would be in this group.

Resistance exercise training appears to be a promising intervention to improve the health of those living with insulin treated diabetes, particularly those who are older. However we have a limited understanding of what form such a resistance training programme might take, how and to what extent it will improve health, and how that might differ between those with and without diabetes.

Aims and objectives

For peer review only - http://bmjopen.bmj.com/site/about/guidelines.xhtml

The purpose of this feasibility trial is to characterise the physical function, cardiovascular health, and the health and wellbeing of older adults with mild frailty and insulin treated diabetes, and to test the feasibility of conducting a trial of resistance training in improving these parameters. The study has two parts: 1) to compare the above parameters against an age, gender, and frailty matched non-diabetes control group, 2) to test the feasibility and acceptability and efficacy of regular resistance exercise as a modality to improve health outcomes in older people with insulin treated diabetes.

Methods and analysis

Trial design

This is a single-centre interventional parallel group feasibility randomised controlled trial conducted in Newcastle-upon-Tyne, England. Thirty participants with insulin treated diabetes and mild frailty, and thirty without diabetes will be randomised 1:1 to the intervention group, which is a 4-week programme of supervised resistance exercise training, or to the control group; to carry on with any usual activity as normal. All participants will be aged ≥60. The current version of the protocol is v2.1.

For peer review only - http://bmjopen.bmj.com/site/about/guidelines.xhtml

The funder (Wellcome Trust) and sponsor (Newcastle-upon-Tyne Hospitals NHS Foundation Trust) will have no role in the study design, conduct, data analysis, results interpretation, or writing.

Exclusion and inclusion criteria

Inclusion criteria for diabetes group (n=30):

- Adults ≥60 years
- Type 1 diabetes OR type 2 diabetes treated with exogenous insulin
- BMI <30 in participants with type 2 diabetes
- Rockwood Clinical Frailty Score of 3 or 4

Inclusion criteria for non-diabetes group (n=30):

- Adults ≥60 years
- Rockwood Clinical Frailty Score of 3 or 4

Exclusion criteria for all groups:

- History of myocardial infarction, stroke, renal failure, severe hypertension, or

For peer review only - http://bmjopen.bmj.com/site/about/guidelines.xhtml

liver disease in the last 12 months

- Unsuitable for the intervention due to limiting musculoskeletal problems
- Inability to give written informed consent

Identification, recruitment, and consent procedures

Potential participants will be identified through the following methods: by their treating clinician who is a clinical member of the research team, in clinic at the Newcastle Diabetes Centre (applicable to those with diabetes only); via poster adverts in GP practices and other secondary care clinics; GP practice database searches facilitated by the North East and North Cumbria Clinical Research Network; via social media; and via the Newcastle United Foundation charity.

A participant information sheet will be sent to potential participants. Informed written consent will be given and eligibility confirmed by a member of the research team. Potential participants will then be screened with the Rockwood Clinical Frailty Score,

For peer review only - http://bmjopen.bmj.com/site/about/guidelines.xhtml

by either their treating diabetes clinician (where applicable) or by a member of the research team. The study will take place from December 2020 to September 2022.

Study procedures

Initial procedures

Participants with diabetes, and participants without, will be age, gender, and frailty matched by the research team to ensure a similar population profile between these two groups.

All 60 participants will undergo the following blood/cardiovascular, physical tests, and patient reported outcome measures at the Newcastle Clinical Research Facility, at the start of the trial and after the four-week intervention/control period:

Blood and cardiovascular :

a) resting blood pressure

b) a 15 ml blood sample will be taken via venepuncture for the quantification of:

For peer review only - http://bmjopen.bmj.com/site/about/guidelines.xhtml

HbA1c, blood lipid profile, inflammatory cytokines by routine hospital clinical chemistry or Newcastle Laboratories. 5 ml will be used to assess counts of endothelial progenitor cells, by flow cytometry as previously described, for a deeper investigation of vascular health in this patient group.[34]

Physical function :

a) Body composition: height, weight, waist circumference, % body fat, and % fat free mass using bioelectrical impedance analysis (SECA 515 Body Composition Analyser).
b) Isometric strength: a torque and strain gauge will be used to assess the force capability of the participants' lower limbs. This test involves maximally extending the leg against an immovable strain gauge, this allows for the calculation of peak force, and time-course changes in force.
c) Handgrip strength: a digital handgrip dynamometer will be used to assess the maximal grip strength of the participants dominant and non-dominant hands.
d) Gait speed: using digital timing gates, the participants will be required to complete three 4m walking tests, to assess the normal walking speed of the

For peer review only - http://bmjopen.bmj.com/site/about/guidelines.xhtml

participants.

e) Timed Sit to stand: participants will sit on a chair and complete 5 stand and sit movements without use of the arms.

Patient reported outcome measures :

For all participants:

a) Health related quality of life: the Short Form-36

For participants with diabetes only:

b) Problem Areas in Diabetes (PAID) scale

c) Hypo Fear Scale (HFS)

Clinical history:

For all participants, information on:

a) Comorbid disease

b) Current medications, including changes in medications during the trial period

c) Weight loss

For peer review only - http://bmjopen.bmj.com/site/about/guidelines.xhtml

d) Exhaustion

e) Physical activity levels

For participants with diabetes only, information on:

a) Insulin regimen

b) Glucose monitoring (self-report)

c) Serious hypoglycaemic episodes over the past 12 months.

For peer review only

Randomisation

After completing the initial testing procedures, all participants will be randomised in a 1:1 ratio to either the intervention group (4-week supervised resistance training programme), or the control group. This will consist of 15 (of the 30) people living with diabetes, and 15 (of the 30) nondiabetic controls. See Figure 1.

Figure 1 here

For peer review only - http://bmjopen.bmj.com/site/about/guidelines.xhtml

Randomisation will be carried out by an individual at Newcastle University who is independent of the study team. Randomisation will be done in a GCP-compliant manner using a web-based randomisation system (http://www.randomization.com/). This will ensure that an equal number of participants from both the diabetes and non-diabetes group are assigned to the intervention and control groups.

Intervention

The intervention is a four-week, semi-structured resistance exercise training programme, designed to increase muscle mass and strength. Training will be carried out at participants' most convenient public gym. The programme involves 2-3 short sessions, per week, for each of the four weeks. Weeks 1 and 2 will be fully supervised by a member of the research team. In week 3, participants will be asked to train alone in one of the sessions, and in week 4, they will train fully independently. A four-week programme has been selected to assess feasibility and acceptability to participants, similar to previous feasibility work carried out in our

For peer review only - http://bmjopen.bmj.com/site/about/guidelines.xhtml

team with older adults.[32] The programme is not designed to induce changes in any physical or clinical outcomes.

Resistance exercise training programme design:

Following extensive explanation and demonstration of proper exercise technique. For each exercise, resistance is increased until momentary failure occurs within 10 repetitions. One repetition maximum (1RM) is estimated using a prediction equation based on using the variables of 'load lifted' and 'number of repetitions completed'. [35] This method has been previously demonstrated as a valid approach for estimating 1RM in older adults.[36]

The following exercise sessions will be completed weekly for 4 weeks:

Session 1: Leg press, leg extension, leg curl, leg adduction, calf raises, chest press, shoulder press, lateral pull down, lateral raises

Repetitions: 8-12 at 70% 1RM, Sets per exercise: 3, recovery between sets: 2 minutes

For peer review only - http://bmjopen.bmj.com/site/about/guidelines.xhtml

Session 2: Leg press, single-leg half leg press, chest press, shoulder press, seated row

Repetitions: 5-8 at 85% 1RM, Sets per exercise: 3, recovery between sets: 4 minutes or feeling recovered.

Session 3: Leg press, leg extension, leg curl, leg adduction, chest press, shoulder press, lateral pull down, lateral raises

Repetitions: 12–15 at 60% 1RM, Sets per exercise: 3, recovery between sets: 2 minutes

Control arm

Participants randomised to the control arm will be asked to carry on with normal daily activities, without any changes to any exercise they might do.

Blinding

For peer review only

For peer review only - http://bmjopen.bmj.com/site/about/guidelines.xhtml

Blinding will not be possible for participants as the intervention involves undertaking a supervised exercise resistance training programme, with the control arm undertaking no additional exercise other than any usual level of activity. The clinical team, and the research staff responsible for analysing quantitative outcomes, will be blinded to treatment allocation. Research staff responsible for supervising the resistance training, and the qualitative aspects of the study, will not be blinded.

Qualitative process evaluation

We will use qualitative methods to develop an in-depth understanding of participant perceptions and experiences on the following topics: age and frailty, physical activity, living with diabetes (where relevant), barriers and facilitators to participating in the study and resistance training more generally, and views on the resistance training programme (where relevant). Topic guides are provided in Supplemental Data. Prior to the intervention commencing, one semi-structured interview will be conducted with up to 20 participants. These will be split equally between the intervention group and control group, and between those with and without diabetes. We will then interview up to 10 intervention group participants after the four-week training programme, to

For peer review only - http://bmjopen.bmj.com/site/about/guidelines.xhtml

understand their views and experiences of the programme and perceived impact on their health and wellbeing. We will also offer participants the option of taking part in this interview as a one-off procedure, without taking part in the trial, should the trial be severely impacted by the COVID-19 global pandemic. We expect each interview to last 45-60 minutes. Interviews will take place at a time and location most suitable for the participants, either face to face or over the telephone.

Patient and Public Involvement

Involvement of the public and stakeholders in the early stages of this study confirmed that the potential impact of exercise on blood glucose control, especially hypoglycaemia, is a common concern for people living with diabetes. Study design was enhanced by capturing the views of several patients with insulin treated diabetes. They reflected on the relevance and importance of the study, study documentation and approach, and potential dissemination strategies for the public. Two PPI members are actively involved in this trial, influencing design and conduct. Their input will be supported according to INVOLVE guidance.

For peer review only - http://bmjopen.bmj.com/site/about/guidelines.xhtml

Study outcomes

Feasibility outcomes

The primary aim of this study is acceptability and feasibility of procedures for recruitment and retention, randomization, and adherence and fidelity to the resistance training programme intervention. Recruitment rates will be calculated as the rate of invited participants who are eligible, who subsequently provide informed consent. Attrition rates will be measured, defined by discontinuation of the resistance training intervention and/or loss to follow up measurement for both conditions. Reasons for attrition will be explored qualitatively. Acceptance, adherence and fidelity to the resistance training intervention will be monitored by the research team, who will measure session attendance, participant following intervention instructions >75% of the time, and participant self-report. Pre- and post-intervention qualitative interviews will be used to assess the acceptability of the resistance training intervention, influences on diabetes self-management where applicable, and wellbeing more generally, at baseline and at 5 weeks. Information about adverse

For peer review only - http://bmjopen.bmj.com/site/about/guidelines.xhtml

events will be collected for the intervention group.

Secondary outcomes

Clinical and physical outcomes

1. Body composition measured using height, weight, waist circumference, percentage body fat, and percentage fat-free mass using bioelectrical impedance analysis at baseline and 5 weeks

2. Isometric strength measured using a torque and strain gauge at baseline and 5 weeks

3. Handgrip strength measured using a digital handgrip dynamometer at baseline and 5 weeks

4. Gait speed measured using three 4m walking tests on digital timing gates at baseline and 5 weeks

5. Timed sit to stand, measured using five sit-to-stand movements at baseline and 5 weeks

6. Cardiovascular health measured using resting blood pressure, HbA1c, blood lipid

For peer review only - http://bmjopen.bmj.com/site/about/guidelines.xhtml

profile, and inflammatory cytokines at baseline and 5 weeks

7. Instances of hypoglycaemia, measured weekly from baseline, obtained using patient self-report.

Sample size calculation

This is a feasibility study with no existing data to draw upon to inform a meaningful sample size calculation. We have selected a sample size in line with previous guidance on feasibility studies[37], and data on key outcomes collected during this trial will inform the sample size calculation for a larger efficacy trial in future.

Data collection and management

Data will be collected by DW, RS, and GT, members of the research team. Quantitative data on blood, cardiovascular, and physical function tests will be gathered using a tailored case report form. Qualitative data, including non-participant observations of the training programme, will be recorded with a voice recording device alongside written field notes.

For peer review only - http://bmjopen.bmj.com/site/about/guidelines.xhtml

Data analysis plan

Quantitative analysis

This is a feasibility study to inform a larger trial and no hypothesis testing will be conducted. Consequently, quantitative data analysis will be descriptive. Mean, SD, range, and 95% CI will be assessed on all quantitative data to assess response rates, numbers of individuals consented and randomised, retention rate, fidelity to the intervention, and participation in the training programme and qualitative interviews. The same descriptive methods will be used to report questionnaire and assessment data at baseline and five weeks. Statistical analyses will be conducted using IBM SPSS Statistics v22 software.

Qualitative analysis

Qualitative data (generated by interviews) will be analysed for thematic content. This approach is both inductive (data interrogated to answer research questions but themes allowed to 'emerge' from the data) and iterative (data collection and analysis

For peer review only - http://bmjopen.bmj.com/site/about/guidelines.xhtml

occurring simultaneously). All interviews will be audio recorded and transcribed verbatim. Data analysis will involve a process of organising the data, descriptive coding, interpretive coding, writing and theorising. Data will be managed using a qualitative computer software package (NVivo v11).

Initially we will seek to understand each participant group (intervention and control), then we will explore similarities and differences across each group. Throughout this process, the constant comparative method of analysis will be used, with an iterative process of data collection and analysis. This will allow identification of initial themes and ideas from the data to be explored in more depth in subsequent interviews, and allows data from different participants to be compared and contrasted, such as intervention vs control participants, participants with type 1 or 2 diabetes, with different levels of frailty and so on. Deviant cases will be actively sought throughout the analysis, and emerging ideas and themes modified in response.

Monitoring and trial management

A data monitoring committee has not been convened given the small size and feasibility focus of this trial. The Trial Management Group will provide trial oversight

For peer review only - http://bmjopen.bmj.com/site/about/guidelines.xhtml

and monitor any safety issues that arise.

Ethics and dissemination

Ethical approval was obtained from the UK Health Research Authority (ref: 20/NE/0178, North East - Newcastle & North Tyneside 2 Research Ethics Committee). This trial is sponsored by the Newcastle-upon-Tyne Hospitals NHS Foundation Trust (ref: 9144).

The report from the clinical trial will be used for publication and oral presentation at scientific meetings. The trial investigators aim to publish the results in writing in clinically relevant open access journals. A summary of findings will also be distributed to our Patient and Public Involvement group.

The trial was registered with an International Standard Randomised Controlled Trials Number (ISRCTN13193281) on 15/07/2020.

Discussion

The health benefits of regular physical exercise for those with, and without, insulin

For peer review only - http://bmjopen.bmj.com/site/about/guidelines.xhtml

treated diabetes are numerous. Many with diabetes choose not to participate in physical activity, often due to fear of hypoglycaemia, or limited knowledge of exercise types and regimens.[38] There is emerging evidence that resistance exercise can be doubly beneficial to older adults with diabetes: it is an exercise modality which appears to carry less risk of hypoglycaemia than other forms of exercise, and has the potential to limit age-related physical deterioration exacerbated by diabetes, such as sarcopenia. However we have a limited understanding of what form such a resistance training programme might take, how and to what extent it will improve health, how it might impact hypoglycaemia, and how outcomes might differ between those with and without diabetes. In the EXPLODE study we will test the acceptability of one resistance exercise training programme, and gather data on how the programme influences the health of older people with insulin treated diabetes. Should this feasibility study generate positive data and demonstrate participant acceptability, we intend to carry out a pilot randomised controlled trial of the same resistance training intervention over a longer duration.

In summary, EXPLODE is a single centre feasibility randomised parallel group trial

For peer review only - http://bmjopen.bmj.com/site/about/guidelines.xhtml

investigating whether resistance exercise training has the potential to improve the health of older adults living with insulin treated diabetes. It will also provide us with information on the acceptability of the resistance training programme and any required design amendments to a future larger pilot.

Author contributions

RS contributed to research design and drafted the manuscript. DW, JS, GT, and MDW contributed to research design and revision of the manuscript. All authors approved the final version of the manuscript to be published. DW is responsible for the integrity of the work as a whole.

Funding statement

This study is being supported by the Wellcome Trust, by a Wellcome Trust Small Grant (grant number: N/A) to DW.

Competing interests

None declared.

For peer review only - http://bmjopen.bmj.com/site/about/guidelines.xhtml

Figure legends

Figure 1. Consolidated Standards of Reporting Trials (CONSORT) diagram.

References

1. Ogurtsova K, da Rocha Fernandes JD, Huang Y, et al. IDF Diabetes Atlas: Global estimates for the prevalence of diabetes for 2015 and 2040. Diabetes Research and Clinical Practice 2017;**128**:40-50.
2. Diabetes UK. Diabetes Prevalence 2019 2019 [Available from: https://www.diabetes.org.uk/professionals/position-statements-reports/statistics/diabetes-prevalence-2019.
3. NHS Digital. National Diabetes Audit Report 1: Care Processes and Treatment Targets, 2018-19 2019 [Available from: https://digital.nhs.uk/data-and-information/publications/statistical/national-diabetes-audit/report-1--care-processes-and-treatment-targets-2018-19-short-report.
4. Ringborg A, Lindgren P, Yin D, et al. Time to insulin treatment and factors associated with insulin prescription in Swedish patients with type 2 diabetes. Diabetes & metabolism 2010;**36**(3):198-203.
5. Home P, Riddle M, Cefalu WT, et al. Insulin Therapy in People With Type 2 Diabetes: Opportunities and Challenges? Diabetes Care 2014;**37**(6):1499-508.
6. Stedman M, Lunt M, Davies M, et al. Cost of hospital treatment of type 1 diabetes (T1DM) and type 2 diabetes (T2DM) compared to the non-diabetes population: a detailed economic evaluation. BMJ Open 2020;**10**(5):e033231.
7. Rawshani A, Rawshani A, Franzén S, et al. Mortality and Cardiovascular Disease in Type 1 and Type 2 Diabetes. New England Journal of Medicine 2017;**376**(15):1407-18.
8. Lebovitz HE. Insulin: Potential Negative Consequences of Early Routine Use in Patients With Type 2 Diabetes. Diabetes Care 2011;**34**(Supplement 2):S225-S30.
9. Monaco CMF, Gingrich MA, Hawke TJ. Considering Type 1 Diabetes as a Form of Accelerated Muscle Aging. Exercise and Sport Sciences Reviews 2019;**47**(2):98-107.
10. Beaudart C, Zaaria M, Pasleau F, et al. Health outcomes of sarcopenia: a systematic review and meta-analysis. PloS one 2017;**12**(1):e0169548.
11. Batsis JA, Mackenzie TA, Barre LK, et al. Sarcopenia, sarcopenic obesity and mortality in older adults: results from the National Health and Nutrition Examination Survey III. European Journal of Clinical Nutrition 2014;**68**(9):1001-07.
12. Cacciatore F, Testa G, Galizia G, et al. Clinical frailty and long-term mortality in elderly subjects with diabetes. Acta diabetologica 2013;**50**(2):251-60.
13. Hubbard R, Andrew M, Fallah N, et al. Comparison of the prognostic importance of diagnosed diabetes, co-morbidity and frailty in older people. Diabetic Medicine 2010;**27**(5):603-06.
14. Hofbauer LC, Brueck CC, Singh SK, et al. Osteoporosis in patients with diabetes mellitus. Journal of Bone and Mineral Research 2007;**22**(9):1317-28.

For peer review only - http://bmjopen.bmj.com/site/about/guidelines.xhtml

15. Jung H-W, Kim S-W, Ahn S, et al. Prevalence and outcomes of frailty in Korean elderly population: comparisons of a multidimensional frailty index with two phenotype models. PloS one 2014;**9**(2).
16. Vlietstra L, Hendrickx W, Waters DL. Exercise interventions in healthy older adults with sarcopenia: A systematic review and meta-analysis. Australasian Journal on Ageing 2018;**37**(3):169-83.
17. Riddell MC, Zaharieva DP, Tansey M, et al. Individual glucose responses to prolonged moderate intensity aerobic exercise in adolescents with type 1 diabetes: The higher they start, the harder they fall. Pediatric Diabetes 2019;**20**(1):99-106.
18. Douen AG, Ramlal T, Rastogi S, et al. Exercise induces recruitment of the "insulin-responsive glucose transporter". Evidence for distinct intracellular insulin- and exercise-recruitable transporter pools in skeletal muscle. The Journal of biological chemistry 1990;**265**(23):13427-30.
19. Gulve EA, Cartee GD, Zierath JR, et al. Reversal of enhanced muscle glucose transport after exercise: roles of insulin and glucose. The American journal of physiology 1990;**259**(5 Pt 1):E685-91.
20. Bohn B, Herbst A, Pfeifer M, et al. Impact of Physical Activity on Glycemic Control and Prevalence of Cardiovascular Risk Factors in Adults With Type 1 Diabetes: A Cross-sectional Multicenter Study of 18,028 Patients. Diabetes Care 2015;**38**(8):1536-43.
21. Brazeau A-S, Rabasa-Lhoret R, Strychar I, et al. Barriers to physical activity among patients with type 1 diabetes. Diabetes care 2008;**31**(11):2108-09.
22. Turner D, Luzio S, Gray B, et al. Algorithm that delivers an individualized rapid-acting insulin dose after morning resistance exercise counters post-exercise hyperglycaemia in people with Type 1 diabetes. Diabetic Medicine 2016;**33**(4):506-10.
23. Turner D, Luzio S, Gray B, et al. Impact of single and multiple sets of resistance exercise in type 1 diabetes. Scandinavian journal of medicine & science in sports 2015;**25**(1):e99-e109.
24. Yardley JE, Kenny GP, Perkins BA, et al. Resistance Versus Aerobic Exercise. Acute effects on glycemia in type 1 diabetes. Diabetes Care 2013;**36**(3):537-42.
25. Bethell H. Sarcopenia, frailty and exercise. Clinical Medicine 2017;**17**(6):591-91.
26. Phu S, Boersma D, Duque G. Exercise and sarcopenia. Journal of Clinical Densitometry 2015;**18**(4):488-92.
27. Durak EP, Jovanovic-Peterson L, Peterson CM. Randomized crossover study of effect of resistance training on glycemic control, muscular strength, and cholesterol in type I diabetic men. Diabetes Care 1990;**13**(10):1039-43.
28. Ramalho AC, de Lourdes Lima M, Nunes F, et al. The effect of resistance versus aerobic training on metabolic control in patients with type-1 diabetes mellitus. Diabetes Res Clin Pract 2006;**72**(3):271-6.
29. Sigal RJ, Kenny GP, Boulé NG, et al. Effects of aerobic training, resistance training, or both on glycemic control in type 2 diabetes: a randomized trial. Ann Intern Med 2007;**147**(6):357-69.
30. Beckwée D, Delaere A, Aelbrecht S, et al. Exercise Interventions for the Prevention and Treatment of Sarcopenia. A Systematic Umbrella Review. The journal of nutrition, health & aging 2019;**23**(6):494-502.
31. Jadczak AD, Makwana N, Luscombe-Marsh N, et al. Effectiveness of exercise interventions on physical function in community-dwelling frail older people: an umbrella review of systematic reviews. JBI database of systematic reviews and implementation reports 2018;**16**(3):752-75.
32. Granic A, Hurst C, Dismore L, et al. Milk and resistance exercise intervention to improve muscle function in community-dwelling older adults at risk of sarcopenia (MIlkMAN): protocol for a pilot study. BMJ Open 2019;**9**(10):e031048.
33. Sinclair A, Gallagher A. Managing frailty and associated comorbidities in older adults with diabetes: Position Statement on behalf of the Association of British Clinical Diabetologists (ABCD), 2020.

For peer review only - http://bmjopen.bmj.com/site/about/guidelines.xhtml

34. West DJ, Campbell MD, Gonzalez JT, et al. The inflammation, vascular repair and injury responses to exercise in fit males with and without Type 1 diabetes: an observational study. Cardiovascular diabetology 2015;**14**(1):1-7.
35. Brzycki M. Strength testing—predicting a one-rep max from reps-to-fatigue. Journal of physical education, recreation & dance 1993;**64**(1):88-90.
36. Knutzen KM, Brilla LR, Caine D. Validity of 1RM prediction equations for older adults. The Journal of Strength & Conditioning Research 1999;**13**(3):242-46.
37. Lancaster GA, Dodd S, Williamson PR. Design and analysis of pilot studies: recommendations for good practice. Journal of evaluation in clinical practice 2004;**10**(2):307-12.
38. Hasan S, Shaw SM, Gelling LH, et al. Exercise modes and their association with hypoglycemia episodes in adults with type 1 diabetes mellitus: a systematic review. BMJ Open Diabetes Research & Care 2018;**6**(1):e000578.

For peer review only

For peer review only - http://bmjopen.bmj.com/site/about/guidelines.xhtml

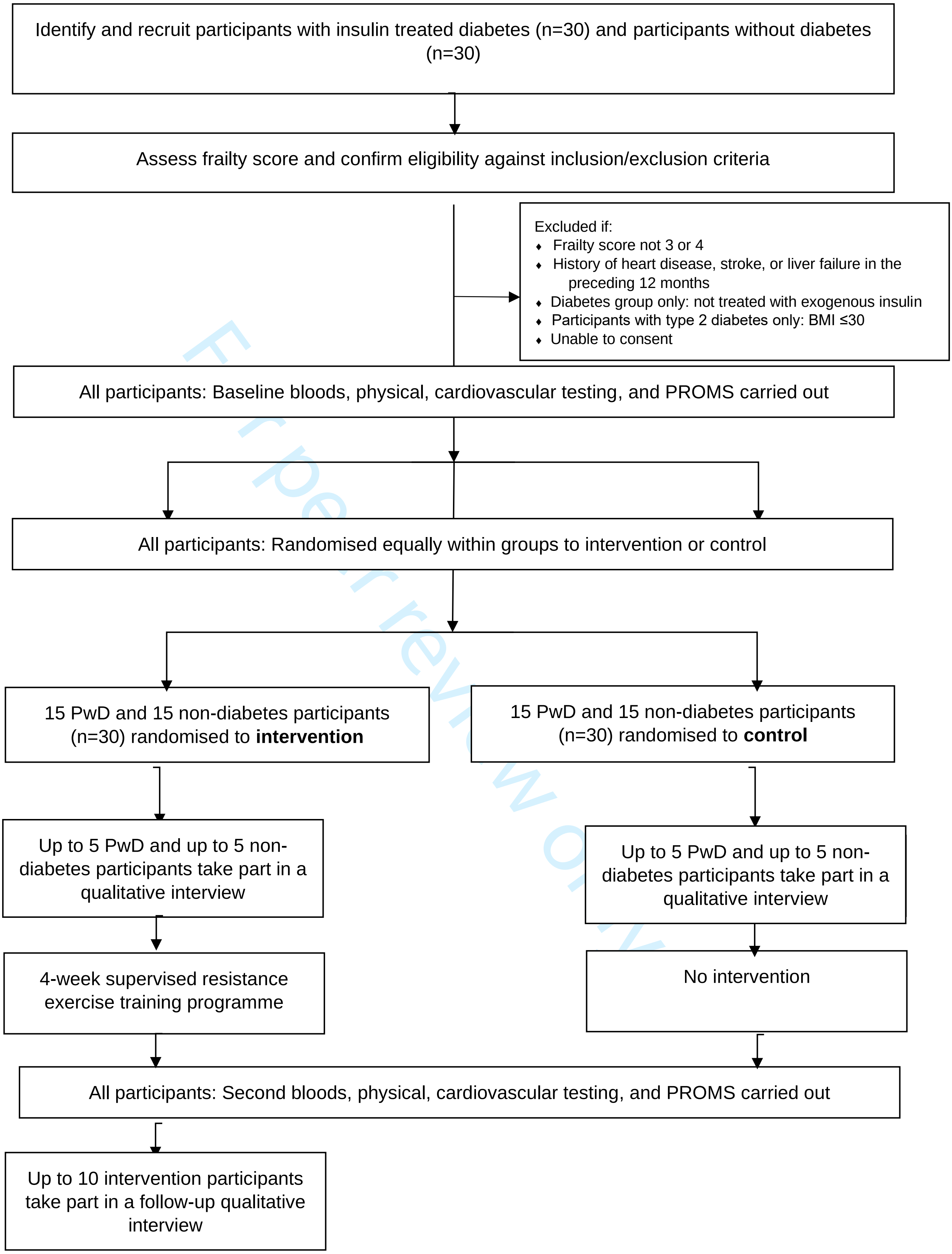

For peer review only - http://bmjopen.bmj.com/site/about/guidelines.xhtml

BMJ Open

EXercise to Prevent frailty and Loss Of independence in insulin treated older people with DiabetEs (EXPLODE): protocol for a feasibility randomised controlled trial (RCT)

Journal:	*BMJ Open*
Manuscript ID	bmjopen-2021-048932.R1
Article Type:	Protocol
Date Submitted by the Author:	07-Sep-2021
Complete List of Authors:	Stocker, Rachel; Newcastle University<br>Shaw, James; Newcastle University<br>Taylor, Guy S; Newcastle University<br>Witham, Miles; Newcastle University, NIHR Newcastle Biomedical Research Centre<br>West, Daniel J; Newcastle University, Population Health Sciences Institute
**Primary Subject Heading**:	Diabetes and endocrinology
Secondary Subject Heading:	Geriatric medicine, Qualitative research, Sports and exercise medicine
Keywords:	DIABETES & ENDOCRINOLOGY, SPORTS MEDICINE, Clinical trials < THERAPEUTICS, GERIATRIC MEDICINE

SCHOLARONE™
Manuscripts

I, the Submitting Author has the right to grant and does grant on behalf of all authors of the Work (as defined in the below author licence), an exclusive licence and/or a non-exclusive licence for contributions from authors who are: i) UK Crown employees; ii) where BMJ has agreed a CC-BY licence shall apply, and/or iii) in accordance with the terms applicable for US Federal Government officers or employees acting as part of their official duties; on a worldwide, perpetual, irrevocable, royalty-free basis to BMJ Publishing Group Ltd ("BMJ") its licensees and where the relevant Journal is co-owned by BMJ to the co-owners of the Journal, to publish the Work in this journal and any other BMJ products and to exploit all rights, as set out in our licence.

The Submitting Author accepts and understands that any supply made under these terms is made by BMJ to the Submitting Author unless you are acting as an employee on behalf of your employer or a postgraduate student of an affiliated institution which is paying any applicable article publishing charge ("APC") for Open Access articles. Where the Submitting Author wishes to make the Work available on an Open Access basis (and intends to pay the relevant APC), the terms of reuse of such Open Access shall be governed by a Creative Commons licence – details of these licences and which Creative Commons licence will apply to this Work are set out in our licence referred to above.

Other than as permitted in any relevant BMJ Author's Self Archiving Policies, I confirm this Work has not been accepted for publication elsewhere, is not being considered for publication elsewhere and does not duplicate material already published. I confirm all authors consent to publication of this Work and authorise the granting of this licence.

For peer review only

EXercise to Prevent frailty and Loss Of independence in insulin treated older people with DiabetEs (EXPLODE): protocol for a feasibility randomised controlled trial (RCT)

Rachel Stocker[1]†, James A Shaw[2], Guy S Taylor[1], Miles D Witham[2], Daniel J West[1]†

† indicates joint corresponding author

[1] Population Health Sciences Institute, Faculty of Medical Sciences, Newcastle University

[2] Translational and Clinical Research Institute, Faculty of Medical Sciences, Newcastle University

Corresponding authors: Dr Rachel Stocker, rachel.stocker@newcastle.ac.uk and Dr Daniel West, daniel.west@newcastle.ac.uk

Abstract

For peer review only - http://bmjopen.bmj.com/site/about/guidelines.xhtml

Introduction

There are 3.9m people in the UK with diabetes. Sarcopenia, increased frailty, and loss of independence are often unappreciated complications of diabetes. Resistance exercise shows promise in reducing these complications in older adult diabetes patients. The aim of this feasibility randomised controlled trial is to (1) characterise the physical function, cardiovascular health, and the health and wellbeing of older adults with mild frailty with/without diabetes treated with insulin, (2) to understand the feasibility and acceptability of a four-week resistance exercise training programme in improving these parameters for those with diabetes, (3) to test the feasibility of recruiting and randomising the diabetic participant group to a trial of resistance training.

Methods and analysis

Thirty adults aged ≥60 years with insulin-treated diabetes mellitus (type 1 or 2), and thirty without, all with mild frailty (3-4 on the Rockwood Frailty Scale) will be recruited. All will complete blood, cardiovascular, and physical function testing. Only the diabetic group will then proceed into the trial itself. They will be randomised 1:1 to a 4-week semi-supervised resistance training programme, designed to increase

For peer review only - http://bmjopen.bmj.com/site/about/guidelines.xhtml

For peer review only

muscle mass and strength, or to usual care, defined as their regular physical activity, for 4 weeks. This group will then repeat testing. Primary outcomes include recruitment rate, attrition rate, intervention fidelity and acceptability, and adherence to the training programme. A subset of participants will be interviewed before and after the training programme to understand experiences of resistance training, impact on health, and living with diabetes (where relevant) as they have aged. Analyses will include descriptive statistics and qualitative thematic analysis.

Ethics and dissemination

The North East-Newcastle & North Tyneside 2 Research Ethics Committee (20/NE/0178) approved the study. Outputs will include feasibility data to support funding applications for a future definitive trial, conference and patient and public involvement presentations, and peer-reviewed publications.

Trial registration

Current Controlled Trials: ISRCTN13193281.

For peer review only - http://bmjopen.bmj.com/site/about/guidelines.xhtml

Strengths and limitations of this study

- This is a novel study using mixed-methods to examine the feasibility of carrying out a larger trial of a gym-based resistance exercise training programme with older, mildly frail adults with insulin-treated diabetes.
- A series of in-depth qualitative interviews will generate a better understanding of the barriers and facilitators to resistance-based exercise for this important patient group, where early intervention is key.
- This study is not intended to be a definitive trial, however, a mixed-methods approach will explore the impact of the training programme on various clinical outcomes and exercise experiences.
- As the study is limited to the North East of England, the sociodemographic characteristics of participants may differ to the population of the wider United Kingdom and other Western countries.

For peer review only - http://bmjopen.bmj.com/site/about/guidelines.xhtml

Introduction

Background

There are around 425 million people with diabetes worldwide, and by 2040 it is predicted that 1 in 10 people globally with have diabetes.[1] In the UK there are around 4 million people living with diabetes[2] (90% Type 2, 8% Type 1, 2% other) with a further 1 million with undiagnosed diabetes. The prevalence of diabetes increases sharply with age, with 17.4% of those aged over 65 having diabetes, compared to 2% of those aged 16 to 44 in England.[3] All of those with type 1 diabetes require insulin treatment, and most of those with type 2 diabetes will eventually also require insulin treatment.[4 5] Diabetes represents >10% of the NHS budget in direct treatment costs.[6] Modern advances in diabetes treatment mean that people with diabetes are living longer[7], even with the presence of the micro- and macrovascular disease associated with long-term diabetes.

With an ageing population of people living with diabetes, it is important that strategies for improving both health, quality of life, and reducing treatment burden

For peer review only - http://bmjopen.bmj.com/site/about/guidelines.xhtml

are identified. Long-term insulin treatment for diabetes is, however, associated with detrimental effects to health including hypoglycaemia and weight gain[8], and may adversely affect muscle health.[9] Sarcopenia in particular leads to an increased risk of frailty, falls, physical disability, chronic metabolic disease, and mortality.[10 11] The prevalence of frailty in older people with diabetes has been reported as ~32–48%, which is significantly higher than that of 5–10% in older persons without diabetes.[12 13] Recent studies have also demonstrated an increased risk of osteoporosis and fracture in older people with diabetes, compared to age matched non-diabetes controls.[14 15] Thus long-term diabetes and long-term insulin treatment, hypoglycaemia and age related physical decline, may carry an additional burden for those living with insulin treated diabetes in later life.

Physical activity and exercise interventions have been shown to improve outcomes associated with frailty and sarcopenia (such as muscle mass, muscle force production, cardiorespiratory fitness).[16] However, these interventions are not straightforward in people with insulin treated diabetes due to the risk of hypoglycaemia.[17] Aerobic exercise increases insulin sensitivity, changes the absorption and action of the injected insulin, and increases metabolic rate

For peer review only - http://bmjopen.bmj.com/site/about/guidelines.xhtml

dramatically.[18 19] Under these conditions, in a person without diabetes, the insulin concentrations would drop dramatically during and after exercise, however, in those with diabetes this is not possible (as the insulin is injected), and thus increases the risk of dangerously low blood glucose occurring. Nearly two-thirds of those with type 1 diabetes do not engage in any physical activity[20], commonly due to fear of exercise-induced hypoglycaemia.[21]

Established insulin-treated non-obese type 2 diabetes shares many characteristics with type 1 diabetes, due to relatively greater insulin deficiency and lower insulin resistance than in type 2 diabetes associated with obesity. This includes intrinsic glucose variability with higher risk of impaired awareness of hypoglycaemia[22 23], including severe events requiring assistance from others in treatment.[24] We hypothesise that mild frailty may have a comparable impact in type 1 diabetes and insulin-treated type 2 diabetes where BMI is <30 kg/m^2 [25,] with potentially comparable impacts of resistance exercise training.

Resistance based exercise, i.e. repeated intense muscle contractions of isolated parts of the body against a fixed load, is associated with less fluctuation in blood

For peer review only - http://bmjopen.bmj.com/site/about/guidelines.xhtml

glucose than aerobic exercise.[26-28] Resistance exercise is a potent stimulus for improving 1) muscle mass, 2) muscle strength and power, 3) bone health, and 4) physical function, such as stair climbing.[29 30] Existing studies have shown resistance training to be a useful exercise modality in older, non-frail type 1 and type 2 diabetes patients.[31-33] It is also the modality of exercise with the most evidence for improving outcomes in older people with sarcopenia or frailty,[34 35] and generally well tolerated by this group.[36] However, data for its use in older people with diabetes is scant. As resistance training potentially carries less risk of blood glucose fluctuation to those with diabetes, due to differing hormonal responses to aerobic exercise, it has potential to be a preferred modality of exercise for this group, and may help sustain long-term engagement.[26-28]

Given the increasing ageing diabetes population, and the increased risk of sarcopenia and frailty in this group, it is important to provide lifestyle related interventions, such as resistance training, to improve the quality of life of older people living with diabetes.[37] At present there is limited information on the physical function of older people with diabetes compared to those without diabetes, as well as how acceptable or feasible a resistance training intervention would be in this group.

For peer review only - http://bmjopen.bmj.com/site/about/guidelines.xhtml

Resistance exercise training appears to be a promising intervention to improve the health of those living with insulin treated diabetes, particularly those who are older. However we have a limited understanding of what form such a resistance training programme might take, and how and to what extent it will improve health.

Aims and objectives

The purpose of this baseline case-control descriptive observational study and subsequent feasibility trial is to characterise the physical function, cardiovascular health, and the health and wellbeing of older people with mild frailty and with/without insulin-treated diabetes, and to test the feasibility of conducting a trial of resistance training in improving these parameters, and the acceptability of regular resistance exercise as a modality to improve health outcomes in older people with insulin treated diabetes.. .

Methods and analysis

Study design

This is a single-centre interventional feasibility randomised controlled trial with an

For peer review only - http://bmjopen.bmj.com/site/about/guidelines.xhtml

associated baseline case-control descriptive observational component, and a qualitative and process evaluation component, conducted in Newcastle-upon-Tyne, England. Thirty participants with insulin treated diabetes and mild frailty, and thirty without diabetes will be recruited. All participants will be aged ≥60. All participants will undergo blood and physical testing, for the baseline case-control component. The diabetic participants will then go forward into the trial. They will be randomised 1:1 to the intervention group, which is a 4-week programme of supervised resistance exercise training, or to the control group; to carry on with any usual activity as normal. The current version of the protocol is v3.

Exclusion and inclusion criteria

Inclusion criteria for diabetes group (n=30):

- Adults ≥60 years
- Type 1 diabetes OR type 2 diabetes treated with exogenous insulin
- BMI <30 in participants with type 2 diabetes
- Rockwood Clinical Frailty Score of 3 or 4

Inclusion criteria for non-diabetes group (n=30):

For peer review only - http://bmjopen.bmj.com/site/about/guidelines.xhtml

- Adults ≥60 years
- Rockwood Clinical Frailty Score of 3 or 4

Exclusion criteria for all groups:

- History of myocardial infarction, stroke, renal failure, severe hypertension, or liver disease in the last 12 months
- Unsuitable for the intervention due to limiting musculoskeletal problems
- Inability to give written informed consent

Identification, recruitment, and consent procedures

All potential participants will be identified through the following methods: by their treating clinician who is a clinical member of the research team, in clinic at the Newcastle Diabetes Centre (applicable to those with diabetes only); via poster adverts in GP practices and other secondary care clinics; GP practice database searches facilitated by the North East and North Cumbria Clinical Research Network;

For peer review only - http://bmjopen.bmj.com/site/about/guidelines.xhtml

via social media; and via the Newcastle United Foundation charity. All methods have been reviewed and approved by the Health Research Authority and the study sponsor, through their ethical and governance review processes.

A participant information sheet will be sent to potential participants. Informed written consent will be given and eligibility confirmed by a member of the research team. Potential participants will then be screened with the Rockwood Clinical Frailty Score, by either their treating diabetes clinician (where applicable) or by a member of the research team. The study will take place from December 2020 to September 2022.

Study procedures

Initial procedures

All 60 participants will undergo the following blood/cardiovascular, physical tests, and patient reported outcome measures at the Newcastle Clinical Research Facility, at the start of the trial and after the four-week intervention/control period:

For peer review only - http://bmjopen.bmj.com/site/about/guidelines.xhtml

Blood and cardiovascular :

a) resting blood pressure

b) a 15 ml blood sample will be taken via venepuncture for the quantification of: HbA1c, blood lipid profile, inflammatory cytokines by routine hospital clinical chemistry or Newcastle Laboratories. 5 ml will be used to assess counts of endothelial progenitor cells, by flow cytometry as previously described, for a deeper investigation of vascular health in this patient group.[38]

Physical function :

a) Body composition: height, weight, waist circumference, % body fat, and % fat free mass using bioelectrical impedance analysis (SECA 515 Body Composition Analyser).

b) Isometric strength: a torque and strain gauge will be used to assess the force capability of the participants' lower limbs. This test involves maximally extending the leg against an immovable strain gauge, this allows for the calculation of peak force, and time-course changes in force.

c) Handgrip strength: a digital handgrip dynamometer will be used to assess the

For peer review only - http://bmjopen.bmj.com/site/about/guidelines.xhtml

maximal grip strength of the participants dominant and non-dominant hands.

d) Gait speed: using digital timing gates, the participants will be required to complete three 4m walking tests, to assess the normal walking speed of the participants.

e) Timed Sit to stand: participants will sit on a chair and complete 5 stand and sit movements without use of the arms.

Patient reported outcome measures :

For all participants:

a) Health related quality of life: the Short Form-36

For participants with diabetes only:

b) Problem Areas in Diabetes (PAID) scale

c) Hypo Fear Scale (HFS)

Clinical history:

For all participants, information on:

For peer review only - http://bmjopen.bmj.com/site/about/guidelines.xhtml

a) Comorbid disease

b) Current medications, including changes in medications during the trial period

c) Weight loss

d) Exhaustion

e) Physical activity levels (using the International Physical Activity Questionnaire, short form)

For participants with diabetes only, information on:

a) Insulin regimen

b) Glucose monitoring (self-report)

c) Serious hypoglycaemic episodes over the past 12 months.

Randomisation

After completing the initial testing procedures, the diabetic group (n=30) will be randomised in a 1:1 ratio to either the intervention group (4-week supervised

For peer review only - http://bmjopen.bmj.com/site/about/guidelines.xhtml

resistance training programme), or the control group. See Figure 1.

Figure 1 here

Randomisation of the diabetic group will take place at the end of the baseline visit. Randomisation will be done in a GCP-compliant manner using a web-based randomisation system (http://www.randomization.com/). The allocation sequence will be prepared by individuals who will remain independent of the study team to preserve allocation concealment. The randomisation code sequence will not be accessible by the study team until after the trial analysis is complete.

Intervention

The intervention is a four-week, semi-structured resistance exercise training programme, designed to increase muscle mass and strength. Training will be carried out at participants' preferred public gym, and facilitated by a trained member of the research team. The programme involves 2-3 sessions lasting less than one hour

For peer review only - http://bmjopen.bmj.com/site/about/guidelines.xhtml

each, per week, for each of the four weeks. Weeks 1 and 2 will be fully supervised by a member of the research team. In week 3, participants will be asked to train alone in one of the sessions, and in week 4, they will train fully independently. A four-week programme has been selected to assess feasibility and acceptability to participants, similar to previous feasibility work carried out in our team with older people.[36] The programme is not designed to induce changes in any physical or clinical outcomes.

The trial will be carried out once all relevant Covid-19 restrictions have been lifted in England. The research team will also adhere to Covid-19 standard operating protocols specified by the sponsor.

Resistance exercise training programme design:

Following extensive explanation and demonstration of proper exercise technique. For each exercise, resistance is increased until momentary failure occurs within 10 repetitions. One repetition maximum (1RM) is estimated using a prediction equation based on using the variables of 'load lifted' and 'number of repetitions completed'. [39]

For peer review only - http://bmjopen.bmj.com/site/about/guidelines.xhtml

This method has been previously demonstrated as a valid approach for estimating 1RM in older people.[40]

The following exercise sessions will be completed weekly for 4 weeks:

Session 1: Leg press, leg extension, leg curl, leg adduction, calf raises, chest press, shoulder press, lateral pull down, lateral raises

Repetitions: 8-12 at 70% 1RM, Sets per exercise: 3, recovery between sets: 2 minutes

Session 2: Leg press, single-leg half leg press, chest press, shoulder press, seated row

Repetitions: 5-8 at 85% 1RM, Sets per exercise: 3, recovery between sets: 4 minutes or feeling recovered.

Session 3: Leg press, leg extension, leg curl, leg adduction, chest press, shoulder

For peer review only - http://bmjopen.bmj.com/site/about/guidelines.xhtml

press, lateral pull down, lateral raises

Repetitions: 12–15 at 60% 1RM, Sets per exercise: 3, recovery between sets: 2 minutes

Control arm

Participants randomised to the control arm will be asked to carry on with normal daily activities, without any changes to any exercise they might do.

Blinding

Blinding will not be possible for participants as the intervention involves undertaking a supervised exercise resistance training programme, with the control arm undertaking no additional exercise other than any usual level of activity. The clinical team, and the research staff responsible for analysing quantitative outcomes, will be blinded to treatment allocation. Research staff responsible for supervising the resistance training, and the qualitative aspects of the study, will not be blinded.

For peer review only - http://bmjopen.bmj.com/site/about/guidelines.xhtml

Qualitative process evaluation

We will use qualitative methods to develop an in-depth understanding of participant perceptions and experiences on the following topics: age and frailty, physical activity, living with diabetes (where relevant), barriers and facilitators to participating in the study and resistance training more generally, and views on the resistance training programme (where relevant). Topic guides are provided in Supplemental Data. Prior to the intervention commencing, one semi-structured interview will be conducted with up to 20 participants. These will be split equally between the intervention group and control group, and between those with and without diabetes. We will then interview up to 10 intervention group participants after the four-week training programme, to understand their views and experiences of the programme and perceived impact on their health and wellbeing. We will also offer participants the option of taking part in this interview as a one-off procedure, without taking part in the trial, should the trial be severely impacted by the COVID-19 global pandemic. We expect each interview to last 45-60 minutes. Interviews will take place at a time and location most suitable for the participants, either face to face or over the telephone. All participants will be approached for interview in order of recruitment. We anticipate that the sample sizes

For peer review only - http://bmjopen.bmj.com/site/about/guidelines.xhtml

described will allow data saturation, and interviews will cease once no new semantic codes are identified from our concurrent thematic analysis of these data (code saturation).[41]

Patient and Public Involvement

Involvement of the public and stakeholders in the early stages of this study confirmed that the potential impact of exercise on blood glucose control, especially hypoglycaemia, is a common concern for people living with diabetes. Study design was enhanced by capturing the views of several patients with insulin treated diabetes. They reflected on the relevance and importance of the study, study documentation and approach, and potential dissemination strategies for the public. Two PPI members are actively involved in this trial, influencing design and conduct. Their input will be supported according to INVOLVE guidance.

Study outcomes

Feasibility outcomes

For peer review only - http://bmjopen.bmj.com/site/about/guidelines.xhtml

The primary aim of this study is acceptability and feasibility of procedures for recruitment and retention, randomization, and adherence and fidelity to the resistance training programme intervention. We will use a traffic light approach, where green = proceed without modification; amber = proceed but with modification, red = unrealistic to proceed without major modification. Recruitment rates will be calculated as the rate of invited participants who are eligible, who subsequently provide informed consent. Green = >50% recruitment, amber = 25-50% recruitment, red = <25% recruitment. Attrition rates will be measured, defined by discontinuation of the resistance training intervention and/or loss to follow up measurement for both conditions. Green = <10% attrition, amber = 10-20% attrition, red = >20% attrition. Reasons for attrition will be explored qualitatively. Acceptance, adherence and fidelity to the resistance training intervention will be monitored by the research team, who will measure session attendance: green = >90% attendance, amber = 75-90% attendance, red = <75% attendance, plus participant following intervention instructions >75% of the time, and participant self-report. Pre- and post-intervention qualitative interviews will be used to assess the acceptability of the resistance training intervention, influences on diabetes self-management where applicable, and

For peer review only - http://bmjopen.bmj.com/site/about/guidelines.xhtml

wellbeing more generally, at baseline and at 5 weeks. Information about adverse events will be collected for the intervention group.

Secondary outcomes

Clinical and physical outcomes

1. Body composition measured using height, weight, waist circumference, percentage body fat, and percentage fat-free mass using bioelectrical impedance analysis at baseline and 5 weeks

2. Isometric strength measured using a torque and strain gauge at baseline and 5 weeks

3. Handgrip strength measured using a digital handgrip dynamometer at baseline and 5 weeks

4. Gait speed measured using three 4m walking tests on digital timing gates at baseline and 5 weeks

5. Timed sit to stand, measured using five sit-to-stand movements at baseline and 5 weeks

For peer review only - http://bmjopen.bmj.com/site/about/guidelines.xhtml

6. Cardiovascular health measured using resting blood pressure, HbA1c, blood lipid profile, and inflammatory cytokines at baseline and 5 weeks

7. Instances of hypoglycaemia, measured weekly from baseline, obtained using patient self-report.

Sample size calculation

This is a feasibility study with no existing data to draw upon to inform a meaningful sample size calculation. We have selected a sample size in line with previous guidance on feasibility studies[42], and data on key outcomes collected during this trial will inform the sample size calculation for a larger efficacy trial in future.

Data collection and management

Data will be collected by DW, RS, and GT, members of the research team. Quantitative data on blood, cardiovascular, and physical function tests will be gathered using a tailored case report form. Qualitative data, including non-participant observations of the training programme, will be recorded with a voice recording

For peer review only

For peer review only - http://bmjopen.bmj.com/site/about/guidelines.xhtml

device alongside written field notes.

Data analysis plan

Quantitative analysis

This is a feasibility study to inform a larger trial and no hypothesis testing will be conducted. Consequently, quantitative data analysis will be descriptive. Mean, SD, range, and 95% CI will be assessed on all quantitative data to assess response rates, numbers of individuals consented and randomised, retention rate, fidelity to the intervention, and participation in the training programme and qualitative interviews. The same descriptive methods will be used to report questionnaire and assessment data at baseline and five weeks. Statistical analyses will be conducted using IBM SPSS Statistics v22 software.

Qualitative analysis

Qualitative data (generated by interviews) will be analysed for thematic content. This approach is both inductive (data interrogated to answer research questions but

For peer review only - http://bmjopen.bmj.com/site/about/guidelines.xhtml

themes allowed to ‘emerge’ from the data) and iterative (data collection and analysis occurring simultaneously). All interviews will be audio recorded and transcribed verbatim. Data analysis will involve a process of organising the data, descriptive coding, interpretive coding, writing and theorising. Data will be managed using a qualitative computer software package (NVivo v11).

Initially we will seek to understand each participant group (intervention and control), then we will explore similarities and differences across each group. Throughout this process, the constant comparative method of analysis will be used, with an iterative process of data collection and analysis. This will allow identification of initial themes and ideas from the data to be explored in more depth in subsequent interviews, and allows data from different participants to be compared and contrasted, such as intervention vs control participants, participants with type 1 or 2 diabetes, with different levels of frailty and so on. Deviant cases will be actively sought throughout the analysis, and emerging ideas and themes modified in response.

Monitoring and trial management

A data monitoring committee has not been convened given the small size and

For peer review only - http://bmjopen.bmj.com/site/about/guidelines.xhtml

feasibility focus of this trial. The Trial Management Group will provide trial oversight and monitor any safety issues that arise.

Ethics and dissemination

Ethical approval was obtained from the UK Health Research Authority (ref: 20/NE/0178, North East - Newcastle & North Tyneside 2 Research Ethics Committee). This trial is sponsored by the Newcastle-upon-Tyne Hospitals NHS Foundation Trust (ref: 9144).

The report from the clinical trial will be used for publication and oral presentation at scientific meetings. The trial investigators aim to publish the results in writing in clinically relevant open access journals. A summary of findings will also be distributed to our Patient and Public Involvement group.

The trial was registered with an International Standard Randomised Controlled Trials Number (ISRCTN13193281) on 15/07/2020.

Discussion

For peer review only - http://bmjopen.bmj.com/site/about/guidelines.xhtml

The health benefits of regular physical exercise for those with, and without, insulin treated diabetes are numerous. Many with diabetes choose not to participate in physical activity, often due to fear of hypoglycaemia, or limited knowledge of exercise types and regimens.[43] There is emerging evidence that resistance exercise can be doubly beneficial to older people with diabetes: it is an exercise modality which appears to carry less risk of hypoglycaemia than other forms of exercise, and has the potential to limit age-related physical deterioration exacerbated by diabetes, such as sarcopenia. However we have a limited understanding of what form such a resistance training programme might take, how and to what extent it will improve health, how it might impact hypoglycaemia, and how outcomes might differ between those with and without diabetes. In the EXPLODE study we will test the acceptability of one resistance exercise training programme, and gather data on how the programme influences the health of older people with insulin treated diabetes. Should this feasibility study generate positive data and demonstrate participant acceptability, we intend to carry out a pilot randomised controlled trial of the same resistance training intervention over a longer duration.

For peer review only - http://bmjopen.bmj.com/site/about/guidelines.xhtml

In summary, EXPLODE is a single centre feasibility randomised parallel group trial investigating whether resistance exercise training has the potential to improve the health of older people living with insulin treated diabetes. It will also provide us with information on the acceptability of the resistance training programme and any required design amendments to a future larger pilot.

Author contributions

RS contributed to research design and drafted the manuscript. DW, JS, GT, and MDW contributed to research design and revision of the manuscript. All authors approved the final version of the manuscript to be published. DW is responsible for the integrity of the work as a whole.

Funding statement

This study is being supported by the Wellcome Trust, by a Wellcome Trust Small Grant (grant number: N/A) to DW.

The funder (Wellcome Trust) and sponsor (Newcastle-upon-Tyne Hospitals NHS Foundation Trust) will have no role in the study design, conduct, data analysis,

For peer review only - http://bmjopen.bmj.com/site/about/guidelines.xhtml

results interpretation, or writing.

Competing interests

None declared.

Figure legends

Figure 1. Consolidated Standards of Reporting Trials (CONSORT) diagram.

References

1. Ogurtsova K, da Rocha Fernandes JD, Huang Y, et al. IDF Diabetes Atlas: Global estimates for the prevalence of diabetes for 2015 and 2040. *Diabetes Research and Clinical Practice* 2017;128:40-50. doi: 10.1016/j.diabres.2017.03.024

2. Diabetes UK. Diabetes Prevalence 2019 2019 [Available from: https://www.diabetes.org.uk/professionals/position-statements-reports/statistics/diabetes-prevalence-2019 accessed 28/09/2020.

For peer review only - http://bmjopen.bmj.com/site/about/guidelines.xhtml

3. NHS Digital. National Diabetes Audit Report 1: Care Processes and Treatment Targets, 2018-19 2019 [Available from: https://digital.nhs.uk/data-and-information/publications/statistical/national-diabetes-audit/report-1--care-processes-and-treatment-targets-2018-19-short-report accessed 28/09/2020.

4. Ringborg A, Lindgren P, Yin D, et al. Time to insulin treatment and factors associated with insulin prescription in Swedish patients with type 2 diabetes. *Diabetes & metabolism* 2010;36(3):198-203.

5. Home P, Riddle M, Cefalu WT, et al. Insulin Therapy in People With Type 2 Diabetes: Opportunities and Challenges? *Diabetes Care* 2014;37(6):1499-508. doi: 10.2337/dc13-2743

6. Stedman M, Lunt M, Davies M, et al. Cost of hospital treatment of type 1 diabetes (T1DM) and type 2 diabetes (T2DM) compared to the non-diabetes population: a detailed economic evaluation. *BMJ Open* 2020;10(5):e033231. doi: 10.1136/bmjopen-2019-033231

7. Rawshani A, Rawshani A, Franzén S, et al. Mortality and Cardiovascular Disease in Type 1 and Type 2 Diabetes. *New England Journal of Medicine* 2017;376(15):1407-18. doi: 10.1056/NEJMoa1608664

For peer review only - http://bmjopen.bmj.com/site/about/guidelines.xhtml

8. Lebovitz HE. Insulin: Potential Negative Consequences of Early Routine Use in Patients With Type 2 Diabetes. *Diabetes Care* 2011;34(Supplement 2):S225-S30. doi: 10.2337/dc11-s225

9. Monaco CMF, Gingrich MA, Hawke TJ. Considering Type 1 Diabetes as a Form of Accelerated Muscle Aging. *Exercise and Sport Sciences Reviews* 2019;47(2):98-107. doi: 10.1249/jes.0000000000000184

10. Beaudart C, Zaaria M, Pasleau F, et al. Health outcomes of sarcopenia: a systematic review and meta-analysis. *PloS one* 2017;12(1):e0169548.

11. Batsis JA, Mackenzie TA, Barre LK, et al. Sarcopenia, sarcopenic obesity and mortality in older adults: results from the National Health and Nutrition Examination Survey III. *European Journal of Clinical Nutrition* 2014;68(9):1001-07. doi: 10.1038/ejcn.2014.117

12. Cacciatore F, Testa G, Galizia G, et al. Clinical frailty and long-term mortality in elderly subjects with diabetes. *Acta diabetologica* 2013;50(2):251-60.

13. Hubbard R, Andrew M, Fallah N, et al. Comparison of the prognostic importance of diagnosed diabetes, co-morbidity and frailty in older people. *Diabetic Medicine* 2010;27(5):603-06.

For peer review only - http://bmjopen.bmj.com/site/about/guidelines.xhtml

14. Hofbauer LC, Brueck CC, Singh SK, et al. Osteoporosis in patients with diabetes mellitus. *Journal of Bone and Mineral Research* 2007;22(9):1317-28.

15. Jung H-W, Kim S-W, Ahn S, et al. Prevalence and outcomes of frailty in Korean elderly population: comparisons of a multidimensional frailty index with two phenotype models. *PloS one* 2014;9(2)

16. Vlietstra L, Hendrickx W, Waters DL. Exercise interventions in healthy older adults with sarcopenia: A systematic review and meta-analysis. *Australasian Journal on Ageing* 2018;37(3):169-83. doi: 10.1111/ajag.12521

17. Riddell MC, Zaharieva DP, Tansey M, et al. Individual glucose responses to prolonged moderate intensity aerobic exercise in adolescents with type 1 diabetes: The higher they start, the harder they fall. *Pediatric Diabetes* 2019;20(1):99-106. doi: 10.1111/pedi.12799

18. Douen AG, Ramlal T, Rastogi S, et al. Exercise induces recruitment of the "insulin-responsive glucose transporter". Evidence for distinct intracellular insulin- and exercise-recruitable transporter pools in skeletal muscle. *The Journal of biological chemistry* 1990;265(23):13427-30. [published Online First: 1990/08/15]

For peer review only - http://bmjopen.bmj.com/site/about/guidelines.xhtml

19. Gulve EA, Cartee GD, Zierath JR, et al. Reversal of enhanced muscle glucose transport after exercise: roles of insulin and glucose. *The American journal of physiology* 1990;259(5 Pt 1):E685-91. doi: 10.1152/ajpendo.1990.259.5.E685 [published Online First: 1990/11/01]

20. Bohn B, Herbst A, Pfeifer M, et al. Impact of Physical Activity on Glycemic Control and Prevalence of Cardiovascular Risk Factors in Adults With Type 1 Diabetes: A Cross-sectional Multicenter Study of 18,028 Patients. *Diabetes Care* 2015;38(8):1536-43. doi: 10.2337/dc15-0030

21. Brazeau A-S, Rabasa-Lhoret R, Strychar I, et al. Barriers to physical activity among patients with type 1 diabetes. *Diabetes care* 2008;31(11):2108-09.

22. Yun JS, Park YM, Han K, et al. Association between BMI and risk of severe hypoglycaemia in type 2 diabetes. *Diabetes Metab* 2019;45(1):19-25. doi: 10.1016/j.diabet.2018.03.006 [published Online First: 2018/04/06]

23. van Meijel LA, de Vegt F, Abbink EJ, et al. High prevalence of impaired awareness of hypoglycemia and severe hypoglycemia among people with insulin-treated type 2 diabetes: The Dutch Diabetes Pearl Cohort. *BMJ Open*

For peer review only - http://bmjopen.bmj.com/site/about/guidelines.xhtml

Diabetes Research & Care 2020;8(1):e000935. doi: 10.1136/bmjdrc-2019-000935

24. Horii T, Oikawa Y, Kunisada N, et al. Real-world risk of hypoglycemia-related hospitalization in Japanese patients with type 2 diabetes using SGLT2 inhibitors: a nationwide cohort study. *BMJ Open Diabetes Research and Care* 2020;8(2):e001856.

25. Izzo A, Massimino E, Riccardi G, et al. A Narrative Review on Sarcopenia in Type 2 Diabetes Mellitus: Prevalence and Associated Factors. *Nutrients* 2021;13(1) doi: 10.3390/nu13010183 [published Online First: 2021/01/14]

26. Turner D, Luzio S, Gray B, et al. Algorithm that delivers an individualized rapid-acting insulin dose after morning resistance exercise counters post-exercise hyperglycaemia in people with Type 1 diabetes. *Diabetic Medicine* 2016;33(4):506-10.

27. Turner D, Luzio S, Gray B, et al. Impact of single and multiple sets of resistance exercise in type 1 diabetes. *Scandinavian journal of medicine & science in sports* 2015;25(1):e99-e109.

For peer review only

For peer review only - http://bmjopen.bmj.com/site/about/guidelines.xhtml

28. Yardley JE, Kenny GP, Perkins BA, et al. Resistance Versus Aerobic Exercise. *Acute effects on glycemia in type 1 diabetes* 2013;36(3):537-42. doi: 10.2337/dc12-0963

29. Bethell H. Sarcopenia, frailty and exercise. *Clinical Medicine* 2017;17(6):591-91.

30. Phu S, Boersma D, Duque G. Exercise and sarcopenia. *Journal of Clinical Densitometry* 2015;18(4):488-92.

31. Durak EP, Jovanovic-Peterson L, Peterson CM. Randomized crossover study of effect of resistance training on glycemic control, muscular strength, and cholesterol in type I diabetic men. *Diabetes Care* 1990;13(10):1039-43. doi: 10.2337/diacare.13.10.1039 [published Online First: 1990/10/01]

32. Ramalho AC, de Lourdes Lima M, Nunes F, et al. The effect of resistance versus aerobic training on metabolic control in patients with type-1 diabetes mellitus. *Diabetes Res Clin Pract* 2006;72(3):271-6. doi: 10.1016/j.diabres.2005.11.011 [published Online First: 2006/01/13]

33. Sigal RJ, Kenny GP, Boulé NG, et al. Effects of aerobic training, resistance training, or both on glycemic control in type 2 diabetes: a randomized trial.

For peer review only

For peer review only - http://bmjopen.bmj.com/site/about/guidelines.xhtml

Ann Intern Med 2007;147(6):357-69. doi: 10.7326/0003-4819-147-6-200709180-00005 [published Online First: 2007/09/19]

34. Beckwée D, Delaere A, Aelbrecht S, et al. Exercise Interventions for the Prevention and Treatment of Sarcopenia. A Systematic Umbrella Review. *The journal of nutrition, health & aging* 2019;23(6):494-502. doi: 10.1007/s12603-019-1196-8 [published Online First: 2019/06/25]

35. Jadczak AD, Makwana N, Luscombe-Marsh N, et al. Effectiveness of exercise interventions on physical function in community-dwelling frail older people: an umbrella review of systematic reviews. *JBI database of systematic reviews and implementation reports* 2018;16(3):752-75. doi: 10.11124/jbisrir-2017-003551 [published Online First: 2018/03/10]

36. Granic A, Hurst C, Dismore L, et al. Milk and resistance exercise intervention to improve muscle function in community-dwelling older adults at risk of sarcopenia (MIlkMAN): protocol for a pilot study. *BMJ Open* 2019;9(10):e031048. doi: 10.1136/bmjopen-2019-031048

For peer review only - http://bmjopen.bmj.com/site/about/guidelines.xhtml

37. Sinclair A, Gallagher A. Managing frailty and associated comorbidities in older adults with diabetes: Position Statement on behalf of the Association of British Clinical Diabetologists (ABCD), 2020.

38. West DJ, Campbell MD, Gonzalez JT, et al. The inflammation, vascular repair and injury responses to exercise in fit males with and without Type 1 diabetes: an observational study. *Cardiovascular diabetology* 2015;14(1):1-7.

39. Brzycki M. Strength testing—predicting a one-rep max from reps-to-fatigue. *Journal of physical education, recreation & dance* 1993;64(1):88-90.

40. Knutzen KM, Brilla LR, Caine D. Validity of 1RM prediction equations for older adults. *The Journal of Strength & Conditioning Research* 1999;13(3):242-46.

41. Braun V, Clarke V. Reflecting on reflexive thematic analysis. *Qualitative Research in Sport, Exercise and Health* 2019;11(4):589-97.

42. Lancaster GA, Dodd S, Williamson PR. Design and analysis of pilot studies: recommendations for good practice. *Journal of evaluation in clinical practice* 2004;10(2):307-12. doi: 10.1111/j..2002.384.doc.x [published Online First: 2004/06/11]

For peer review only - http://bmjopen.bmj.com/site/about/guidelines.xhtml

43. Hasan S, Shaw SM, Gelling LH, et al. Exercise modes and their association with hypoglycemia episodes in adults with type 1 diabetes mellitus: a systematic review. *BMJ Open Diabetes Research & Care* 2018;6(1):e000578. doi: 10.1136/bmjdrc-2018-000578

For peer review only

For peer review only - http://bmjopen.bmj.com/site/about/guidelines.xhtml

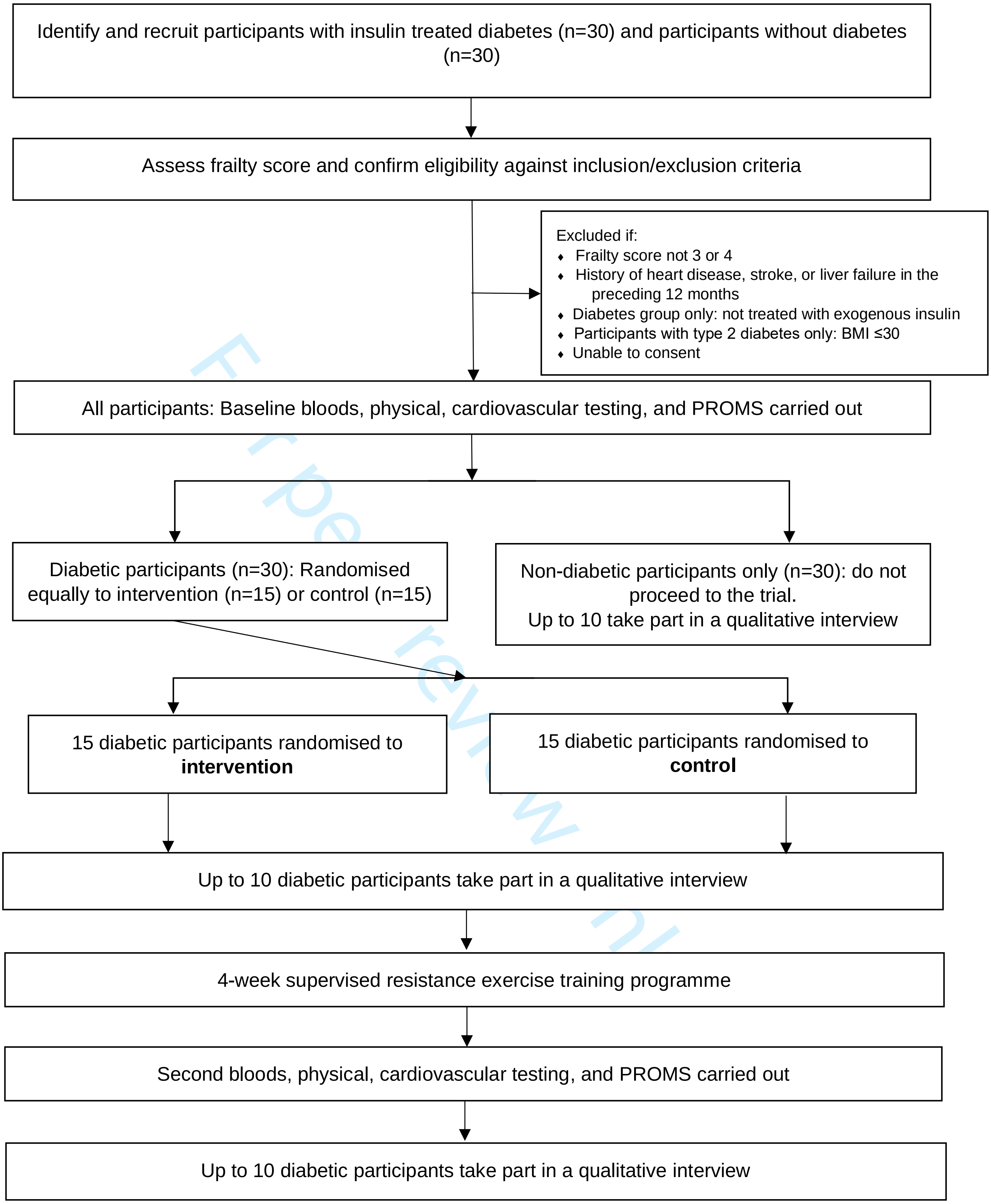
Identify and recruit participants with insulin treated diabetes (n=30) and participants without diabetes (n=30)
Assess frailty score and confirm eligibility against inclusion/exclusion criteria
Excluded if:
Frailty score not 3 or 4
History of heart disease, stroke, or liver failure in the preceding 12 months
Diabetes group only: not treated with exogenous insulin
Participants with type 2 diabetes only: BMI ≤30
Unable to consent
All participants: Baseline bloods, physical, cardiovascular testing, and PROMS carried out
Diabetic participants (n=30): Randomised equally to intervention (n=15) or control (n=15)
Non-diabetic participants only (n=30): do not proceed to the trial.
Up to 10 take part in a qualitative interview
15 diabetic participants randomised to intervention
15 diabetic participants randomised to control
Up to 10 diabetic participants take part in a qualitative interview
4-week supervised resistance exercise training programme
Second bloods, physical, cardiovascular testing, and PROMS carried out
Up to 10 diabetic participants take part in a qualitative interview

CONSORT 2010 checklist of information to include when reporting a pilot or feasibility trial*

Section/Topic	Item No	Checklist item	Reported on page No
**Title and abstract**			
	1a	Identification as a pilot or feasibility randomised trial in the title	1
	1b	Structured summary of pilot trial design, methods, results, and conclusions (for specific guidance see CONSORT abstract extension for pilot trials)	1-2
**Introduction**			
Background and objectives	2a	Scientific background and explanation of rationale for future definitive trial, and reasons for randomised pilot trial	4-6
	2b	Specific objectives or research questions for pilot trial	6-7
**Methods**			
Trial design	3a	Description of pilot trial design (such as parallel, factorial) including allocation ratio	7
	3b	Important changes to methods after pilot trial commencement (such as eligibility criteria), with reasons	n/a – protocol paper
Participants	4a	Eligibility criteria for participants	7-8
	4b	Settings and locations where the data were collected	8-9
	4c	How participants were identified and consented	
Interventions	5	The interventions for each group with sufficient details to allow replication, including how and when they were actually administered	9-11
Outcomes	6a	Completely defined prespecified assessments or measurements to address each pilot trial objective specified in 2b, including how and when they were assessed	15-16
	6b	Any changes to pilot trial assessments or measurements after the pilot trial commenced, with reasons	n/a – protocol paper
	6c	If applicable, prespecified criteria used to judge whether, or how, to proceed with future definitive trial	15-16
Sample size	7a	Rationale for numbers in the pilot trial	17
	7b	When applicable, explanation of any interim analyses and stopping guidelines	n/a – protocol paper
Randomisation:			
Sequence generation	8a	Method used to generate the random allocation sequence	11
	8b	Type of randomisation(s); details of any restriction (such as blocking and block size)	11

For peer review only

Allocation concealment mechanism	9	Mechanism used to implement the random allocation sequence (such as sequentially numbered containers), describing any steps taken to conceal the sequence until interventions were assigned	11
Implementation	10	Who generated the random allocation sequence, who enrolled participants, and who assigned participants to interventions	11
Blinding	11a	If done, who was blinded after assignment to interventions (for example, participants, care providers, those assessing outcomes) and how	13
	11b	If relevant, description of the similarity of interventions	n/a – protocol paper
Statistical methods	12	Methods used to address each pilot trial objective whether qualitative or quantitative	14, 17-18
**Results**			
Participant flow (a diagram is strongly recommended)	13a	For each group, the numbers of participants who were approached and/or assessed for eligibility, randomly assigned, received intended treatment, and were assessed for each objective	n/a – protocol paper
	13b	For each group, losses and exclusions after randomisation, together with reasons	n/a – protocol paper
Recruitment	14a	Dates defining the periods of recruitment and follow-up	n/a – protocol paper
	14b	Why the pilot trial ended or was stopped	n/a – protocol paper
Baseline data	15	A table showing baseline demographic and clinical characteristics for each group	n/a – protocol paper
Numbers analysed	16	For each objective, number of participants (denominator) included in each analysis. If relevant, these numbers should be by randomised group	n/a – protocol paper
Outcomes and estimation	17	For each objective, results including expressions of uncertainty (such as 95% confidence interval) for any estimates. If relevant, these results should be by randomised group	n/a – protocol paper
Ancillary analyses	18	Results of any other analyses performed that could be used to inform the future definitive trial	n/a – protocol paper
Harms	19	All important harms or unintended effects in each group (for specific guidance see CONSORT for harms)	n/a – protocol paper
	19a	If relevant, other important unintended consequences	n/a – protocol paper
**Discussion**			
Limitations	20	Pilot trial limitations, addressing sources of potential bias and remaining uncertainty about feasibility	

For peer review only

Generalisability	21	Generalisability (applicability) of pilot trial methods and findings to future definitive trial and other studies	19
Interpretation	22	Interpretation consistent with pilot trial objectives and findings, balancing potential benefits and harms, and considering other relevant evidence	n/a – protocol paper
	22a	Implications for progression from pilot to future definitive trial, including any proposed amendments	n/a – protocol paper
**Other information**			
Registration	23	Registration number for pilot trial and name of trial registry	2
Protocol	24	Where the pilot trial protocol can be accessed, if available	n/a
Funding	25	Sources of funding and other support (such as supply of drugs), role of funders	20
	26	Ethical approval or approval by research review committee, confirmed with reference number	2

Citation: Eldridge SM, Chan CL, Campbell MJ, Bond CM, Hopewell S, Thabane L, et al. CONSORT 2010 statement: extension to randomised pilot and feasibility trials. BMJ. 2016;355.
*We strongly recommend reading this statement in conjunction with the CONSORT 2010, extension to randomised pilot and feasibility trials, Explanation and Elaboration for important clarifications on all the items. If relevant, we also recommend reading CONSORT extensions for cluster randomised trials, non-inferiority and equivalence trials, non-pharmacological treatments, herbal interventions, and pragmatic trials. Additional extensions are forthcoming: for those and for up to date references relevant to this checklist, see www.consort-statement.org.

For peer review only
